# Direct, Broad-Spectrum Antimicrobial Activity of Ag^+^-Doped Hydroxyapatite against Fastidious Anaerobic Periodontal and Aerobic Dental Bacteria

**DOI:** 10.3390/ma17194688

**Published:** 2024-09-24

**Authors:** Ruibo Hu, Leyi Deng, Xiaoying Hao, Jiadong Chen, Xianfeng Zhou, Nita Sahai

**Affiliations:** 1 School of Polymer Science and Polymer Engineering, The University of Akron, Akron, OH 44325-3909, USA; 2 College of Material Science and Engineering, Qingdao University of Science and Technology, Qingdao 266042, China; 3 Integrated Bioscience Program, The University of Akron, Akron, OH 44325-3909, USA; 4 Department of Geosciences, The University of Akron, Akron, OH 44325-3909, USA; 5 Department of Biology, The University of Akron, Akron, OH 44325-3909, USA

**Keywords:** silver ion, oxidation state, hydroxyapatite, anaerobic bacteria, broad-spectrum antimicrobial

## Abstract

Periodontitis and caries, while seemingly innocuous medical conditions, actually pose significant challenges because of their potential etiology with far more serious conditions. Efficacious treatment is hindered by bacterial antibiotic resistance. Standard AgNPs are ineffective against periodontal anaerobic bacteria, because they require oxidative dissolution to release Ag^+^ ions, which are the actual antimicrobial agents, but oxidation is not possible under anaerobic conditions. Prior studies on Ag-based periodontal antimicrobial materials either did not confirm a silver oxidation state or did not use strictly anaerobic growth media or both, causing spurious antimicrobial efficacy estimates. Here, we prove that silver ion-doped hydroxyapatite nanoparticles (AgHAp NPs) synthesized at various pHs contain an Ag^+^ oxidation state and directly release Ag^+^ even in a strictly anerobic medium. Thus, these AgHAp NPs exhibit direct antimicrobial activity against the fastidious anaerobic Gram-negative periodontal bacterium *Fusobacterium nucleatum* (*F. nucleatum*) and against caries-causing aerobic, Gram-positive bacterium *Streptococcus mutans* (*S. mutans*). The synthesis pH (6–11) correlates inversely with the Ag^+^ content (4.5–0.45 wt %) of AgHAp NPs and, hence, with antimicrobial efficacy, thus providing tunable efficacy for the target application. AgHAp NPs had greater antimicrobial efficacy than Ag^0^-containing AgNPs and were less cytotoxic to the mouse fibroblast L929 cell line. Thus, AgHAp NPs (especially AgHAp7) are superior to AgNPs as effective, broad-spectrum, biocompatible antimicrobials against both anaerobic periodontal and aerobic dental bacteria. AgHAp NP synthesis is also inexpensive and scalable, which are significant factors for treating large global populations of indigent people affected by periodontitis and dental caries.

## 1. Introduction

Bacterially induced oral conditions, including periodontitis and dental caries, pose a significant burden on the global population, affecting approximately 45% according to the World Health Organization [1,2,3]. The prevalence rate is higher in low- and middle-income countries and among the elderly [3,4,5,6]. Periodontitis and caries can lead to various issues, such as discolored teeth, irreversible alveolar bone absorption, and tooth loss, ultimately increasing the risk of developing chronic and lethal diseases, if left untreated [7,8,9]. The gradual progression from gingivitis to periodontitis involves over ten types of bacteria, heightening the risk of conditions including cancer, cardiovascular disease, and inflammatory bowel diseases [2,7,10,11,12,13,14]. However, the high cost of medical treatment in developing countries often render them inaccessible to many patients [5]. Therefore, the development of inexpensive and easily producible antimicrobial materials has become an urgent necessity, and we are motivated to do fundamental research that is socially relevant.

Silver nanoparticles (AgNPs) and their composites have been extensively researched for combating bacterial infections. Various mechanisms have been proposed for the bactericidal action of AgNPs, but recent works have demonstrated that the suggested mechanisms ultimately involve oxidation of Ag^0^ to Ag^+^ with the subsequent release of Ag^+^ ions [15,16,17,18,19]. Therefore, AgNPs are ineffective against anaerobic periodontal bacteria, because the release of Ag^+^ ions is thermodynamically unfavorable under anaerobic conditions [20,21]. Importantly, it was also shown that dissolved Ag^+^ ions, added as soluble AgNO_3_, are directly responsible for killing periodontal anaerobic bacteria [22,23]. A further limitation of AgNPs is that bacteria can develop a resistance to them [17,24].

Hydroxyapatite (HAp; Ca_10_(PO_4_)_6_(OH)_2_) stands as an excellent biocompatible and bioactive mineral because of its similar chemical composition and structure to the mineral constituents found in bone, dentin, and enamel [25] and serves in a wide array of biomedical applications, particularly in orthopedic and dental implants [26,27,28]. Antimicrobial ions, such as Ag^+^, Cu^2+^, and Zn^2+^ associated with Hap, have been explored previously [29,30,31,32,33,34,35,36]; among which, Ag^+^ emerges as the most promising candidate due to its release efficacy across a wide pH range and because of the difficulty for bacteria to develop resistance against Ag^+^ ions [37,38,39,40,41]. However, some limitations existed in previous reports of the Ag^+^-doped HAp NPs as an antimicrobial material. For instance, the oxidation state of Ag^+^ in the HAp crystal structure is not confirmed, with only a few exceptions [42,43,44], or they focus on aerobic bacteria where the culture medium can support Ag^0^ to Ag^+^ oxidation [30,31,33,34], or strictly anaerobic conditions that include L-Cysteine were not reported in the few studies that explored anaerobic bacteria with Ag^+^-HAp NP composites [36,42,45,46].

We were therefore inspired to explore Ag^+^ ion-doped hydroxyapatite nanoparticles (AgHAp NPs) as an antimicrobial material against anaerobic Gram-negative periodontal bacteria *F. nucleatum* and aerobic Gram-positive bacteria *S. mutans*. In addition to this material ideally already containing Ag^+^ ions, thus rendering the antimicrobial efficacy even under anaerobic conditions, it is inexpensive to synthesize even at scale and is also biocompatible.

The goals of the present study were to confirm the oxidation state of silver in AgHAp NPs to determine the efficacy of AgHAp NPs against fastidious periodontal bacterium *F. nucleatum* in a strictly anaerobic medium that contains L-Cys and against aerobic, caries-causing bacterium *S. mutans* and to optimize the Ag content of the AgHAp NPs for these oral infection bacteria. Our previous study demonstrated the broad-spectrum antimicrobial efficacy of AgHAp NPs for orthopedic applications against *E. coli* and *S. epidermidis* and established an inverse relationship of the Ag content in AgHAp with increasing synthesis pH from 9.5–11 [47]. In the present study, we (i) synthesized and characterized AgHAp NPs at pH 6–9.5 to further extend the pH range explored in our previous work [47]; (ii) established the silver oxidation state in the NPs and the distribution of the Ag^+^ ion in the HAp NPs within the surface layer only or throughout the bulk crystal; (iii) determined the antimicrobial efficacy of AgHAp NPs synthesized at various pHs compared to standard AgNPs against *F. nucleatum* and *S. mutans* by the inhibition zone and MIC tests; (iv) tested two anaerobic media conditions, with and without L-Cys, for *F. nucleatum* and one medium for *S. mutans*; (v) estimated the in vitro cytotoxicity towards the mouse fibroblast cell line L-929 compared to AgNPs; and (vi) showed the inhibition zone diameter test as a superior measurement method for anaerobic bacteria compared to the MIC test, consistent with a previous review of the literature [48].

## 2. Materials and Methods

### 2.1. Materials

Ultrapure water (18.2 MΩ·cm) (Barnstead™ GenPure™ xCAD Plus, Thermo Scientific, Rockford, IN, USA) was used for preparing all solutions. The chemicals used in this study include silver nitrate (AgNO_3_, 99.995%, Salt Lake Metals, Salt Lake City, UT, USA) and calcium hydroxide (Ca(OH)_2_, 98%, extra pure, Acros Organics, Morris Plains, NJ, USA). Phosphoric acid (H_3_PO_4_, ACS reagent, ≥85 wt. % in H_2_O), ammonium hydroxide solution (NH_4_OH, ACS reagent, 28–30%), sodium hydroxide solution (NaOH, 50% in H_2_O), and hydrochloric acid (HCl, ACS reagent, 37%) were all purchased from Sigma-Aldrich (St. Louis, MO, USA). Gas Pack Anaerobic Systems, agar, tryptic soy broth (TSB), and brain–heart infusion (BHI) broth were bought from BD (Becton, Dickinson and Co., Sparks, MD, USA). Yeast Extract, vitamin K1, and hemin solutions were bought from Fisher Scientific (Hampton, NH, USA).

### 2.2. AgHAp and HAp Synthesis

The AgHAp NP synthesis method followed our previous work [47]. Briefly, 1.764 g of Ca(OH)_2_ was suspended in 50 mL ultrapure water, followed by stirring for 30 min. After that, 0.204 g AgNO_3_ powder was added into the suspension and stirred for another 30 min, followed by the dropwise addition of a 50 mL portion of 0.3 M H_3_PO_4_ solution. The speed of the dropwise H_3_PO_4_ addition was controlled at 1 mL.min^−1^ to control the particle size. Ammonium hydroxide solution was used to adjust the final pH to 6, 7, 7.5, 8, 8.5, 9.5, or 11, and the final products were labeled as AgHAp6, AgHAp7, AgHAp7.5, AgHAp8, AgHAp8.5, AgHAp9.5, and AgHAp11. The suspensions at different pHs were further stirred overnight (~16 h) and then aged for 1 week. The NPs were washed with ultrapure water at least 5 times, followed by lyophilization (Labconco Freezone 4.5, Kansas City, MO, USA). Pure HAp NPs were also synthesized using the same protocol but without any silver nitrate. All products were ground thoroughly for at least 10 min and stored in a sealed container in the dark before use.

### 2.3. Material Characterization

#### 2.3.1. Morphology and Size

The morphology of NPs was characterized by High-Resolution Transmission Electron Microscopy (HRTEM, Tecnai F20, Hillsboro, OR, USA). A 70% (*v*/*v*) ethanol solution was chosen to disperse the NPs, achieving a concentration of 0.5 mg.mL^−1^. The dispersion was then sonicated for 10 min at 20% amplitude. Subsequently, 2.5 μL of the sonicated suspension was carefully dropped onto Formvar carbon-coated copper grids (300 mesh, Ted Pella, Redding, CA, USA). After a 3-min interval, the excess liquid was gently blotted, and the grids were left overnight to air dry. The particle size distribution was characterized using TEM (JEOL 1230, Peabody, MA, USA), and the photos were analyzed using ImageJ software (version 1.54g). Two replicate grids of each NP type of each sample were prepared for morphological and size analysis, and five spots were images of each grid. The particle size reported was the mean of 10 measurements per NP type, and the error represented the standard deviation from the mean.

#### 2.3.2. Crystal Structure

Crystallinity of the lyophilized powders was characterized by X-ray Diffraction (XRD) (SMART Apex, Bruker, Billerica, MA, USA). A Cu Kα tube operated at 40 kV and 35 mA was applied to generate X-rays. Each NP sample was scanned in the range of 2θ = 15−80° using a step width of 0.01°. Three independent batches (trials) of samples were synthesized and analyzed similarly.

#### 2.3.3. Total Silver Content of AgHAp NPs

AgHAp NP powder was dissolved to 5% (*v*/*v*) nitric acid solution at a NP concertation of 1 mg.mL^−1^. The sample stock was subsequently diluted 10 times before determining the total silver concentration using Inductively Coupled Plasma Optical Emission Spectrometry (ICP-OES) (Agilent 720, Santa Clara, CA, USA). Three replicates of each NP type were analyzed, and three measurements were made on each replicate. The reported Ag content value represents the mean of nine measurements, and the error was calculated as the standard deviation from the mean. Three separate batches (trials) of samples were synthesized and analyzed similarly.

#### 2.3.4. Silver Oxidation State

PHI Quantera SXM-03 Scanning X-ray Photoelectron Spectroscopy (XPS) Microprobe (Physical Electronics, Inc., Chanhassen, MN, USA) was employed to determine the oxidation states of silver in AgHAp7, AgHAp9.5, and AgNPs NPs, as well as for quantitating the relative amounts of each oxidation state of silver relative to the calcium and phosphorus peaks in the AgHAp7 and AgHAp9.5 powder samples. Monochromatic Al Kα (1486.6 eV) radiation edge was utilized, and the electron take-off angle was set at 45°. The pass energies for the survey scan and high-resolution spectra were set at 280 eV and 26 eV, respectively. The beam size was 100 µm. Multipack software Version 9.7.0.1 (Physical Electronics, Chanhassen, MN, USA) was used for peak fitting and quantitative calculations of the tested materials.

### 2.4. Antimicrobial Tests

#### 2.4.1. Bacterial Culture and Growth

Anaerobic Gram-negative bacterium *F. nucleatum* (ATCC 25586) was acquired from MicroBiologics (St. Cloud, MN, USA) in the form of KWIK-STIK swabs, which were activated as per the enclosed instructions. Blood agar plates were prepared following the FDA M20 guidelines [49]. Two different kinds of cell culture liquid medium were prepared for *F. nucleatum* growth. Medium 1 was prepared according to the instructions provided by ATCC-1490 that consisted of brain–heart infusion (BHI) broth (BD, Sparks, MD, USA), 0.5 wt % yeast extract, vitamin K, hemin, and 0.5 g.L^−1^ L-Cysteine [50]. Medium 2 was identical to Medium 1 but did not contain L-Cysteine.

*F. nucleatum* was first cultured in an anaerobic tube under anaerobic conditions at 37 °C in an incubator shaker (Innova 4300, New Brunswick Scientific, Edison, NJ, USA) with shaking at 280 rpm. The anaerobic mixed gas (80% N_2_, 10% H_2_, and 10% CO_2_) was utilized to flush out oxygen during the preparation process of the anaerobic liquid medium, followed by sealing in anaerobic tubes (Chemglass Life Sciences, Vineland, NJ, USA) while inside the Vinyl anaerobic chamber (Coy Laboratory Products, Grass Lake, MI). In addition, BD Gaspack^TM^ EZ pouch anaerobic systems (BD, Sparks, MD, USA) were employed to ensure an anaerobic atmosphere for the agar plates.

*Streptococcus mutans* (*S. mutans*, ATCC 25175) was obtained from the ATCC (American Type Culture Collection, Manassas, VA, USA). Agar and liquid medium consisting of only BHI (“Medium 3”) were prepared following the guidelines of ATCC Medium 44. The *S. mutans* cultures were incubated in Medium 3 under normal atmospheric conditions at 37 °C in an Innova 4300 incubator shaker (New Brunswick Scientific, Edison, NJ, USA) with shaking at 280 rpm.

#### 2.4.2. Inhibition Zone Test

An overnight culture of either *F. nucleatum* or *S. mutans* was used to inoculate 10 mL of each type of liquid growth medium. The cultures were then incubated at 37 °C in the incubator shaker with orbital shaking at 280 rpm until they reached an optical density at 600 nm (OD600) of 0.6. Subsequently, based on the results of colony-forming unit (CFU) counting, the cultures were diluted to obtain a working suspension of approximately 1 × 10^6^ CFU.mL^−1^. Next, 100 μL of the bacterial working suspension was pipetted onto the surface of a FDA M20 blood agar plate for *F. nucleatum* or a BHI agar plate for *S. mutans*.

A cell spreader (Fisher Scientific, Hampton, NH, USA) was used to gently streak the bacterial suspension evenly across the surface of each agar plate. A suspension of NPs at a concentration of 20 mg.mL^−1^ was sonicated for 30 min, and then, 20 μL of the suspension was dropped onto sterilized paper blank disks (Remel, Lenexa, KS, USA). These paper disks were placed onto the surface of the agar plates. The blood agar plates inoculated with *F. nucleatum* were placed into BD EZ pouch systems and incubated at 37 °C for 72 h, and the *S. mutans*-inoculated agar plates were incubated at 37 °C for 72 h in an incubator (Thermo Scientific Forma 3130, Waltham, MA, USA) The diameter of each inhibition zone was measured in two directions using a caliper. The experiments were performed in triplicate. The experiment was repeated for a total of two trials. The diameters reported were the mean of 12 measurements, and the error bar represented the standard deviation from the mean.

#### 2.4.3. Minimum Inhibition Concentration (MIC)

The MIC was determined as the lowest concentration of NPs that inhibited bacterial growth. For *F. nucleatum*, we used Medium 1 with L-Cysteine and Medium 2 without L-Cysteine, while aerobic Medium 3 was used for *S. mutans*. In detail, a volume of 100 μL bacterial working suspension (initial concentration of 1 × 10^6^ CFU.mL^−1^) was dispensed into the wells of a 96-well plate. Then, 100 μL of each type of NP suspended in the bacterial culture medium (initial concentration of 2 mg.mL^−1^ for AgHAp7 and 4 mg.mL^−1^ for AgHAp9.5) was added to each well plate. These well plates were then serially diluted to the desired NP concentrations. Additionally, the negative control medium (NCM), which consisted of only the medium without bacteria or NPs, and negative control bacteria (NCB), consisting of bacteria with a medium but no NPs, were included in other wells of the plate. The 96-well plate was incubated in an incubator (Thermo Scientific Forma 3130, Waltham, MA, USA) at 37 °C for 24 h. The turbidity (OD600) of each plate was measured with a Synergy H1 Hybrid Multi-Mode Reader (BioTek, Winooski, VT, USA), with orbital shaking at 400 rpm before each reading to obtain an even suspension.

The MIC values were determined by identifying that concentration of NPs at which the OD600 value was statistically identical to the OD600 of the negative control. The experiment was repeated in triplicate. The experiment was repeated for a total of three independent trials. The MIC values reported were the mean of 9 measurements, and the error bar represented the standard deviation from the mean.

### 2.5. Silver Complexation

Analytical methods for determining an aqueous Ag^+^ concentration face some challenges [51,52], and ionic conductivity emerges as a reliable method [53]. Ag^+^-L-Cysteine complexation was therefore determined by measuring the Ag^+^ concentration in the absence and presence of L-Cysteine, either aerobically or anaerobically. Two sets of silver nitrate solutions, each with a total volume of 4 mL, were prepared in black polypropylene tubes to achieve the final silver concentrations of 20, 40, 60, 80, and 100 mg.L^−1^ at pH 7 (0.1 M MOPS buffer). MOPS buffer was selected, as it is the least coordinating buffer for Ag^+^ ions [54]. L-Cys was added to one set of solutions to achieve a final concentration of 141.3 mg. L^−1^. In the above procedures, for the anaerobic experiments, solutions were bubbled with N_2_ for 40 min at 60 °C before use in an anaerobic chamber (Vinyl Anaerobic Chambers, Coy Laboratory Products, Grass Lake, MI, USA) equipped with anaerobic mixed gases (95% N_2_ and 5% H_2_).

All aerobic and anaerobic experiments with and without L-Cysteine were left to react for 30 min in the dark. After reaction, the solutions were vortexed, centrifuged (Eppendorf Centrifuge 5810 R) at 2000 rpm for 10 min, and the resulting supernatant was collected for conductivity measurement using a conductivity meter (EC700 Benchtop Conductivity Meter Kit, Apera Instruments, Columbus, OH, USA).

The conductivity tests were conducted in triplicate, with three measurements for each replicate. A total of three independent trials was conducted. The conductivity values reported referred to the average of twenty-seven measurements, and the error bar represented the standard deviation from the mean.

### 2.6. Fibroblast Cell Cytocompatibility

#### 2.6.1. Fibroblast Cell Culture and Growth

Mouse fibroblast cell line L929 (ATCC, Manassas, VA, USA) at passage 9 were cultured in the H-DMEM medium (High-Glucose Dulbecco’s Modified Eagle’s Medium, Gibco) with 10% FBS (fetal bovine serum (HyClone, Thermo Scientific, Rockford, IN, USA) and 1% P/S Solution (Penicillin/Streptomycin 100× solution, HyClone, Thermo Scientific, Rockford, IN, USA). The cultures were stored in a water-jacketed incubator (Forma II, Thermo Scientific) at 37 °C in a 5% CO_2_ atmosphere. The L929 fibroblast cells were harvested by 0.25% EDTA-trypsin solution, and the cell stock concentration was adjusted to 1 × 10^6^ cells.mL^−1^. To seed the L-929 cells, 100 μL of cell stock solution was pipetted into the wells of a 96-well plate and incubated for 48 h at 37 °C (Forma II, Thermo Scientific, USA) to allow the cells to attach and proliferate. After incubation, the growth medium was discarded, and the cells in the well plate were treated with NP suspensions at various concentrations. 

To make a NP stock dilution, NPs were directly suspended in H-DMEM medium to obtain an initial NP concentration of 1.5 mg.mL^−1^. Then, 100 mL of this NP stock solution was pipetted into a second 96-well plate and diluted serially with cell culture medium to obtain the desired NP concentrations. These final NP concentration solutions were added to the cells in the first 96-well plate.

#### 2.6.2. LIVE–DEAD Assay by Fluorescence Microscopy

Intracellular esterase activity in live cells and loss of plasma membrane integrity in dead cells were determined using the fluorescent LIVE (green)/DEAD (red) assay (Invitrogen, Grand Island, NY, USA) to estimate cell viability. After incubating bacteria in 96-well plates with various NP concentrations in growth media for 24 h or 72 h, the media was discarded. A 100 μL staining solution (2 μM calcein AM and 4 μM EthD-1 in PBS) was added, followed by incubation for 10 min, according to the manufacturer’s instructions (Invitrogen, Grand Island, NY, USA). To obtain clear images, staining solutions were aspirated, followed by adding 100 mL PBS buffer. Cells were visualized using an inverted fluorescence microscope (Olympus IX51, Olympus American Inc., Melville, NY, USA). Three replicate wells were used for each condition (NP concentration), and one image per well was recorded.

#### 2.6.3. MTT Assay

Cellular redox potential was used as a measure of metabolic activity to estimate cell viability by the MTT assay (3-(4,5-dimethylthiazol-2-yl)-2,5-diphenyltetrazolium bromide, Sigma-Aldrich, St. Louis, MO, USA). In addition to adding the NP suspensions at various concentrations, pure medium was also added into the 96-well plate to make positive and negative controls. Positive control contains L929 cells, medium, and 30 μL Tween 20 but no NPs, while negative control contains L929 cells and medium but no NPs. Blank samples consisting of only the NP suspensions at various concentrations without any cells were also utilized to minimize errors caused by UV–Vis absorbance by NPs and by the 96-well plates. After incubating at 37 °C for 24 or 72 h in a CO_2_ incubator, 20 μL of MTT solution was added to each well of the plate following a 4-h incubation. Then, the solutions were discarded, and 200 μL of DMSO was added to each well. After 30 min, the optical density at 570 nm was measured using a plate reader (Spark, Tecan, Switzerland) for the Sample, Positive Control, Negative Control, and Blank wells. To account for nanoparticle interference, the OD570 value of the Blank well was subtracted from each other well. Cell viability at each nanoparticle concentration was then calculated using Equation (1).

(1) $$\mathrm{Cell}\;\mathrm{Viability}\;=\frac{\left({\mathrm{OD}570}_{\mathrm{Sample}}\;-\;\mathrm{OD}570_{\mathrm{Positive}}\right)}{(\mathrm{OD}570_{\mathrm{Negative}}\;-\;\mathrm{OD}570_{\mathrm{Positive}})}$$

OD570_Sample_, OD570_Positive_, and OD570_Negative_ represent the optical density of the blank-corrected Sample, Positive Control, and Negative Control, respectively.

The concentration that inhibits the fibroblast cell viability by 50% (IC50) was determined by curve-fitting the dose–response curve using Logistic 3P models. Inverse prediction was selected to calculate the 50% proliferation with a confidence level of 0.95.

The experiment was conducted in triplicate, with three measurements per replicate. The results reported were the mean of nine measurements, and the error bars represented the standard deviation from the mean. The experiment was repeated for a total of three independent trials.

### 2.7. Statistical Analysis

Statistical analysis of the NP size, diameter of the inhibition zone, and MIC data was performed using JMP Pro 16 software. The error represented the standard deviation from the mean. Statistical differences between groups were analyzed using one-way ANOVA for NP size and the Student’s *t*-test for the inhibition zone and MIC data. Statistically significant differences will be indicated by an asterisk in the figures.

## 3. Results and Discussion

### 3.1. Material Characterization

#### 3.1.1. Appearance of HAp and AgHAp NPs

The color of all HAp and AgHAp samples is white, similar to natural teeth, despite the incorporation of silver ions into the HAp crystal (Figure S1A), as opposed to using AgNPs (Figure S1B), which appear black due to a surface oxidized layer, as will be shown below by XPS (Section 3.1.4). This white color offers potential for future oral applications, because improved aesthetics would probably increase patient compliance in application of the antimicrobial treatment.

**Figure 1:**
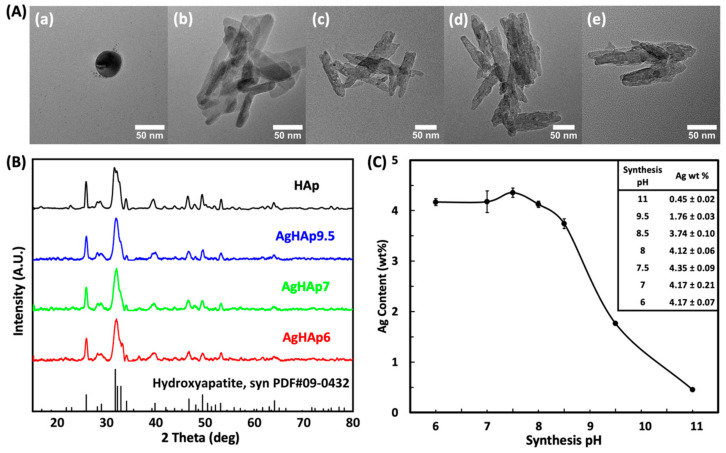
Characterization of Ag0, Hap, and AgHAp NPs. (**A**) TEM images of (**a**) AgNPs, (**b**) HAp, (**c**) AgHAp9.5, (**d**) AgHAp7, and (**e**) AgHAp6; (**B**) XRD patterns of the NPs; (**C**) Total silver content obtained from ICP-OES analysis for the AgHAp NPs synthesized at different pHs. Error bar represents the standard deviation from the mean.

The morphology and size of the AgNPs, HAp, AgHAp9.5, AgHAp7, and AgHAp6 were estimated by TEM (Figure 1A and Figure S2). The AgNPs are spheres with a diameter ~50 nm, whereas HAp and AgHAp NPs present as rod-like particles ~90 nm in length × 20 nm in width. No significant difference in particle size was observed between HAp and all types of AgHAp NPs (Figure S2). Therefore, the physical shape and size of the AgHAp NPs are not affected by introducing silver into the HAp crystals.

In addition, a few very small (1–2 nm diameter) electron-dense dark spots associated with AgHAp NPs are seen occasionally (e.g., Figure 1A(c,d)). These are most likely a small amount of Ag^0^ NPs, as will be seen below from the high-resolution XPS scan results (Section 3.1.4).

#### 3.1.2. Crystal Structure

All the synthesized AgHAp NPs exhibited a single-phase HAp crystal structure, as evidenced by the typical diffraction peak positions that correspond to the standard HAp XRD pattern (PDF #09-0432) (Figure 1B). The formation of a stable Ag^+^-doped HAp single phase crystal is also supported by the absence of diffraction peaks related to pure AgNPs. Thus, the presence of silver ions does not appear to affect the HAp NP crystal structure.

#### 3.1.3. Total Silver Content

As the synthesis pH decreases, the silver content increases until it stabilizes when the pH falls below 7 (Figure 1C and Table S1). The Ag content incorporated into the synthesized AgHAp NPs almost doubled from ~2 wt % to ~4 wt % as the synthesis pH decreased from 9.5 to 7 but did not change significantly when the synthesis pH was further decreased to 6 (Table 1). A similar trend was observed previously of increasing the Ag content of AgHAp NPs with decreasing the synthesis pH from 11 to pH 9.5 (~0.6–~2 wt %, respectively) [47]. This trend can be understood by considering that the Ag^+^ ions can partially substitute for Ca^2+^ ions in the HAp crystal lattice. During AgHAp NP synthesis, the Ag^+^ ions in the alkaline solution (added as AgNO_3_) bind to OH^−^ ions and precipitate as Ag_2_O at basic pHs, thus limiting the available Ag^+^ ions in a solution for uptake into the HAp crystal lattice. The lack of significant difference between AgHAp6 and AgHAp7 can be attributed to the limitation imposed by the initial amount of Ag^+^ ions added during synthesis.

#### 3.1.4. Silver Oxidation State

**Figure 2:**
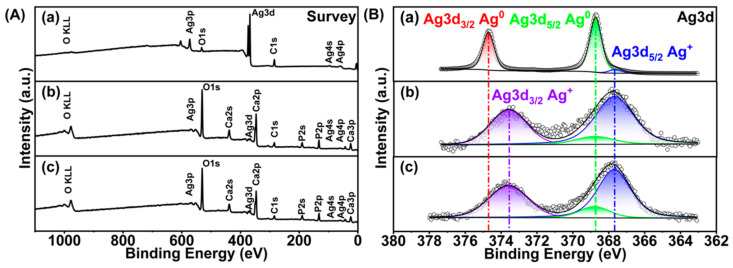
XPS analysis. (**A**) Survey spectrum for (**a**) AgNPs, (**b**) AgHAp9.5, and (**c**) AgHAp7. (**B**) High-resolution scan of Ag3d XPS spectra: (**a**) AgNPs, (**b**) AgHAp9.5, and (**c**) AgHAp7. Open circles: raw spectra; black curve: fitted peak; filled area: fitted component peak.

Because Ag^+^ ion is the actual antimicrobial agent [15,16,22,55], it is critical to establish the oxidation state of silver in AgHAp and AgNPs. Determining the Ag^+^/Ag^0^ ratio and total Ag content will also help us to calculate the stoichiometry of the AgHAp NPs. XPS analysis was employed to analyze the composition of the AgHAp NPs, the oxidation state of silver, and the distribution of the Ag on the surface versus throughout the HAp crystal lattice by combining with the ICP-OES results. The survey scan of XPS analysis showed the presence of Ca, P, O, and C (Figure 2A and Table 1, columns 3–7). The Ag content in atom % obtained by XPS survey scan (Table 1, column 7) is converted to wt % (Table 1, column 8). The resulting Ag contents are very close to those obtained from ICP-OES (Figure 1C), suggesting that the Ag is distributed throughout the AgHAp NPs.

High-resolution XPS scans at the Ag3d edge were employed to quantitate the oxidation state of silver in AgHAp7 and AgHAp9.5 compared to AgNPs as a reference (Figure 2B and Table 2). The Ag3d_5/2_ and Ag3d_3/2_ peaks for Ag^0^ occur at 368.8 eV and 374.7 eV [56] compared to slightly downshifted values of 367.7 and 373.6 eV for Ag^+^ [57]. The phenomenon of a negative binding energy shift of silver ions from its metallic state has been previously reported in multiple studies [58,59] and is caused by initial state factors such as ionic charge and lattice potential [60]. A similar downshifting of energies is observed in our AgHAp NPs compared to AgNPs, suggesting the presence of Ag^+^ ions in the AgHAp NPs (Figure 2B).

To further quantitate the relative amounts of Ag^0^ and Ag^+^ ion in each type of NP, the peaks were deconvoluted, and the area under the peaks was estimated. For Ag^0^ NPs, the area under the peaks at 368.8 eV and 374.7 eV was greater than that under 367.7 and 373.6 eV, indicating that 94% of the silver in the AgNPs was zero-valent silver. A small component of oxidized silver (~6 atom %, Table 2) was also detected in the AgNPs. This reflects the inevitable oxidation of AgNPs to Ag^+^ in a thin surface layer, and this effect makes the NPs appear black (Figure S1B).

The corresponding analysis for AgHAp NPs reveals that the peaks corresponding to Ag^+^ (blue and purple, Figure 2B) are larger than that of the peak corresponding to Ag^0^ (green curve, Figure 2B). Thus, 83–87% silver in AgHAp is present as Ag^+^, with the remainder being Ag^0^ (Table 2).

To calculate the stoichiometric formula (Ca_10-x_Ag_x_(PO_4_)_6_(OH)_2-x_, where x is the Ag mole fraction) in AgHAp NPs, we assume that only Ag^+^ can enter the Ca^2+^ lattice sites. The total Ag was determined by XPS survey scan (Table 1, column 7) and the % Ag^+^ content was provided by high-resolution XPS (Table 2, column 5), which yielded x = 0.03 and x = 0.05, respectively, for AgHAp9.5 and AgHAp7 NPs.

The small amount of Ag^0^ (13–15%, Table 2, column 5) is either present as tiny Ag^0^ NPs (Figure 1A(c,d)) associated with AgHAp NPs or is present in the channels parallel to the crystallographic *c*-axis of AgHAp nanocrystals. Many different types of neutral inorganic and organic molecules, including H_2_O, CO_2_, O_2_, Ar, glycine, acetic acid, and amino-2-ethylphosphate, have been reported to be accommodated in these channels [61,62,63,64,65,66].

In summary, the high-resolution scans of the Ag3d region confirms that the silver is predominantly present as Ag^+^ and is evenly distributed throughout the AgHAp NPs, whereas Ag^0^ predominantly exists in the AgNPs with a thin oxidation surface layer.

### 3.2. Antimicrobial Tests

#### 3.2.1. Inhibition Zone

**Figure 3:**
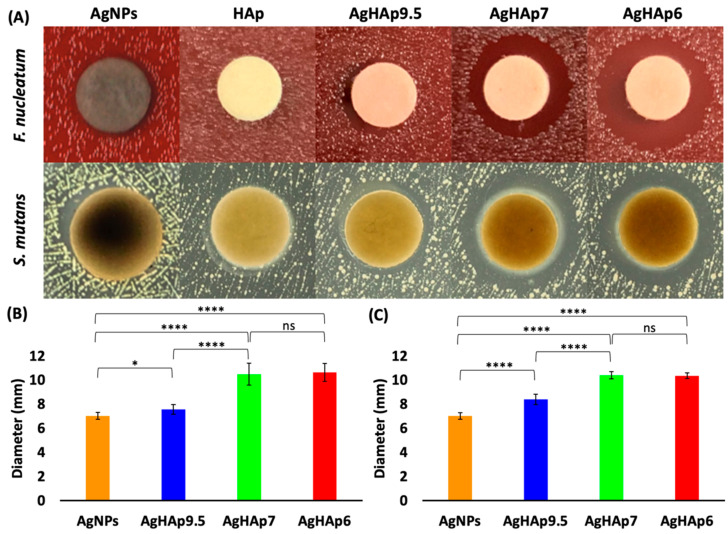
Inhibition zone antimicrobial test results for AgNPs, HAp, AgHAp9.5, AgHAp7, and AgHAp6 against *F. nucleatum* and *S. mutans*. (**A**) Agar plate images for *F. nucleatum* (top panel) and *S. mutans* (middle panel). (**B**,**C**) Inhibition zone diameters against *F. nucleatum* (**B**) and *S. mutans* (**C**). Student *t*-test was selected for statistical analysis (* < 0.05, **** < 0.0001, and ns = no significant difference).

Inhibitory effects of the NPs were found to depend on the silver oxidation state, Ag^+^ content of AgHAp NPs, and bacterial type. In detail, the inhibitory effect of all the AgHAp NPs is greater than that of the AgNPs for both bacteria, indicating the direct action of Ag^+^ ions. The AgNPs did exhibit limited inhibitory effects compared to the “blank” of pure HAp (Figure 3A). Further, the inhibition zone diameter increased from ~7 mm for AgHAp9.5 to ~11 mm for AgHAp7, while no statistical difference was found between AgHAp7 and AgHAp6 (Figure 3A,B). These results are consistent with the trend of increasing the Ag content with the synthesis pH from pure HAp to AgHAp 9.5 to AgHAp7 or AgHAp6 (Table 1). The inhibition zone diameters of all the AgHAp NPs were similar for *F. nucleatum* (Figure 3B and Table S2) and *S. mutans* (Figure 3C and Table S2), except for AgHAp9.5, which was less effective against *F. nucleatum*.

#### 3.2.2. MIC

The MIC values of AgHAp and AgNPs were estimated against the anaerobic Gram-negative bacterium *F. nucleatum* in two growth media (Media 1 and 2) and against the aerobic Gram-positive bacterium *S. mutans* in a third growth medium (Medium 3) (Table 3 and Figure S3). The MIC values for all NPs appeared quite high at first glance, but the values corrected for the actual Ag content were significantly lower by factors of 25-fold and 57-fold, respectively, for AgHAp7 and AgHAp9.5.

Several important trends emerge from the data (Table 3 and Figure S3) that are related to the silver oxidation state in the NPs, total silver content of the AgHAp NPs based on the synthesis pH, anaerobic growth medium effects, and differences in bacterial surface properties. Firstly, the MIC values of all AgHAp NPs were lower than that of all AgNPs for both *F. nucleatum* and *S. mutans*, indicating that AgHAp NPs are generally more effective. The greater antimicrobial efficacy of AgHAp compared to AgNPs against *F. nucleatum* is consistent with the direct release of Ag^+^ ions, which are the actual antimicrobial agent, from the AgHAp samples. In contrast, the AgNPs require oxidative dissolution to release Ag^+^, which is highly unfavorable under anaerobic conditions [20,21]. The less efficacious antimicrobial behavior of AgNPs against *S. mutans* in aerobic Medium 3 compared to that of all AgHAp NPs, however, was unexpected (Figure S3), where Ag^+^ ion release by oxidative dissolution of the AgNPs was expected. A potential reason for this observation is that, although oxidative release of Ag^+^ is more thermodynamically favorable in Medium 3, the reaction rate may be slower than that of direct Ag^+^ release by AgHAp NPs. This slower rate for AgNPs may be related to the surface oxide layer revealed by XPS analysis (Table 2), which passivates the surface against further oxidation. Recently, other studies have also revealed that AgNPs are less effective against aerobic bacteria compared to Ag^+^ ions [67,68].

The second important trend from the MIC results is that the MIC for AgHAp7 < AgHAp9.5 in all media and both bacteria, consistent with the decreasing Ag content of the AgHAp NPs with a synthesis pH.

Significantly, comparing the MIC results in Medium 1 versus 2 revealed the critical role of anaerobic growth medium on the MIC values (Table 3). For AgHAp7, the MIC values against *F. nucleatum* in Medium 1 containing L-Cys are four-fold smaller (MIC = 375 μg.mL^−1^) than those in Medium 2 lacking L-Cys (1500 μg.mL^−1^). At least two effects of L-Cys could be involved that influence the apparent MIC. The presence of L-Cys results in a more reducing growth medium, which promotes healthy bacterial growth compared to a medium that lacks it. This is true even though the experiments with Medium 2 were conducted with meticulous care, including the use of the anaerobic chamber to prepare growth media and to culture cells, as well as the inclusion of BD Gas Packs to scrub traces of O_2_. Thus, Medium 2 may only be a quasi-anaerobic medium, yielding artificially low MIC values due to unhealthy bacterial growth. A second potential effect of L-Cys could be that it acts as a ligand to form complexes with Ag^+^ ions, which depresses the availability of the antimicrobial agent (Ag^+^ ion), which will be tested below in Section 3.3. Both effects may work together to yield high apparent MIC values in Medium 1 compared to 2.

Finally, somewhat more puzzling is the less efficacious antimicrobial behavior of AgHAp9.5 towards *S. mutans* in aerobic conditions (Medium 3) compared to that of AgHAp9.5 towards *F. nucleatum* under quasi-anaerobic conditions (Medium 2). Differences in the bacterial cell surface properties and growth medium effects could potentially underlie this puzzling observation. For instance, Ag^+^ ions are less able to penetrate the thicker peptidoglycan cell wall of Gram-positive *S. mutans* compared to that of Gram-negative *F. nucleatum* [69]. Moreover, Gram-positive bacteria have a less negatively charged membrane than Gram-negative bacteria [70,71,72,73], which could result in a weaker electrostatic attraction of Ag^+^. Finally, *F. nucleatum* bacterial growth was likely less in the absence of L-Cysteine (quasi anaerobic Medium 2), which makes it more susceptible to the antimicrobial AgHAp9.5 NPs than *S. mutans* in Medium 3.

### 3.3. Silver Complexation

**Figure 4:**
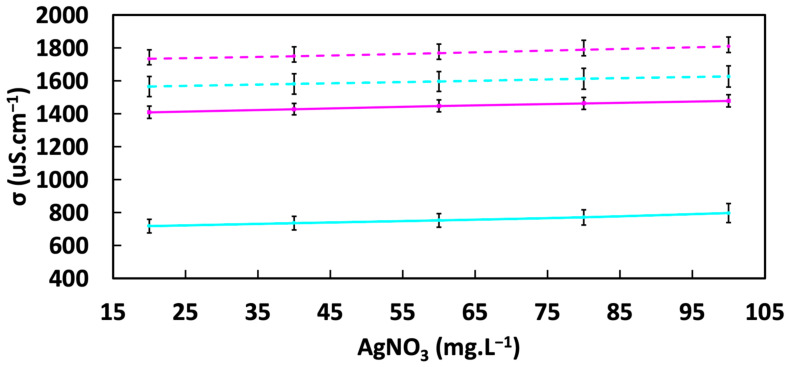
Conductivity test under aerobic (cyan) and anaerobic (magenta) conditions with L-Cysteine (solid line) added and without L-Cysteine (dashed line).

We hypothesized that L-Cys in Medium 1 complexes Ag^+^ ions, thereby decreasing the free Ag^+^ concentration. To test this hypothesis, the concentrations of Ag^+^-bearing solutions were determined at various concentrations in the absence and presence of L-Cysteine, either aerobically or anaerobically. As expected, ionic conductivity increased with the increasing concentration of dissolved AgNO_3_ (Figure 4). The addition of L-Cys significantly reduced the solution’s conductivity under both anaerobic and aerobic conditions. These results support our hypothesis that the concentration of free Ag^+^ ions decreased by interactions with the S atom of the thiol moiety of L-Cysteine, consistent with previous works [53,74,75,76].

The drop in ionic conductivity of the solution upon the addition of L-Cysteine was ~800 μS.cm^−1^ and ~400 μS.cm^−1^, respectively, under aerobic and anaerobic conditions. The larger drop in conductivity under aerobic conditions shows that the complexation of Ag^+^ by L-Cysteine is more favorable under aerobic circumstances. To explain this result, we suggest that Ag^+^ acts as an oxidant in this system, while cysteine functions as a reducing agent. In the case of the thiol group, the oxidation of the group also corresponds to its deprotonation, where the pK_a_ is ~8.3. The presence of O_2_ in the aerobic system enhances the generation of oxidized (deprotonated) sulfur atoms, consequently facilitating the binding of more Ag^+^ ions. The H^+^s released from the oxidation of the thiol group are neutralized by the MOPs buffer, which maintains the pH at 7. Thus, the equilibrium is shifted towards greater thiol group oxidation under aerobic conditions and, consequently, more bonding of free Ag^+^ ions by S atoms compared to the anaerobic system, as reflected in the larger decline in ionic conductivity.

### 3.4. Fibroblast Cell Cytocompatibility

#### 3.4.1. Fibroblast Cell Imaging

**Figure 5:**
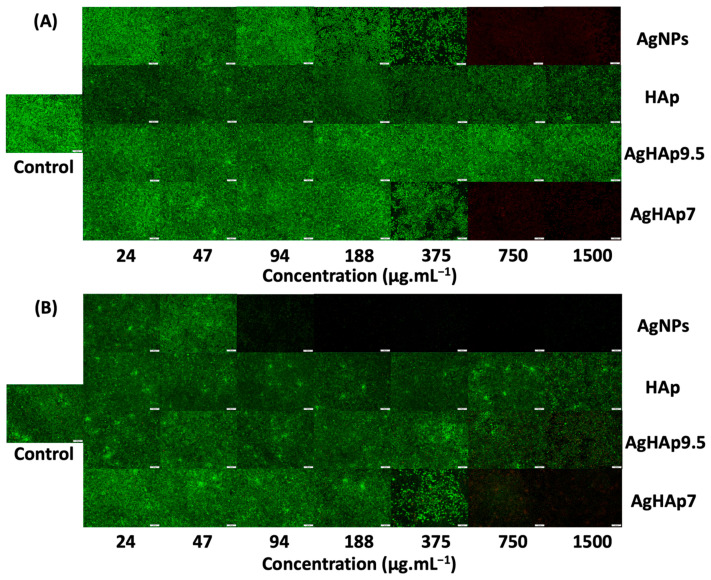
Cell viability by the LIVE/DEAD assay of murine fibroblast L-929 cells in the presence of various concentrations of AgHAp NPs compared to AgNPs and pure HAp NPs on (**A**) Day 1 and (**B**) Day 3. All images were taken at 50× magnification with the same scale bar of 100 µm. Images shown have not been adjusted for differences in contrast.

The LIVE/DEAD assay was used to test the cytotoxicity of NPs to murine fibroblast L-929 cells (Figure 5). On Day 1, the AgNPs showed increasing toxicity, from concentrations of 47 μg.mL^−1^ for AgNPs to 375 μg.mL^−1^ for AgHAp7 NPs and complete cell death at concentrations > 375 μg.mL^−1^ compared to the control system without any NPs (Figure 5A). By Day 3, the AgNPs were highly toxic at concentrations > 47 μg.mL^−1^, and all cells were dead by concentrations ≥ 94 μg.mL^−1^ (Figure 5B).

In the presence of AgHAp7 NPs, a noticeable decrease in cell density was seen at a concentration of ~375 μg.mL^−1^ on Day 1, and nearly all cells were dead at higher concentrations (Figure 5A). By Day 3, some cells were lysed at 375 μg.mL^−1^, though a few live cells were still discernible at 750 μg.mL^−1^, and the cells were practically all dead at 1500 μg.mL^−1^ (Figure 5B). AgHAp9.5 NPs were not toxic to L-929 cells, even at 1500 μg.mL^−1^, and concentrations > 750 μg.mL^−1^ resulted in a few dead cells. The lower cytotoxicity of AgHAp9.5 compared to AgHAp7 is consistent with the lower Ag content of the former.

HAp NPs were completely nontoxic at all concentrations on Day 1, as expected based on HAp’s biocompatibility, and only a small number of dead cells were observed at high concentrations on Day 3. The above results indicate that AgNPs are significantly more cytotoxic to L-929 cells than all the AgHAp NPs, and this effect became even more significant after Day 1.

#### 3.4.2. Fibroblast Cell Viability

**Figure 6:**
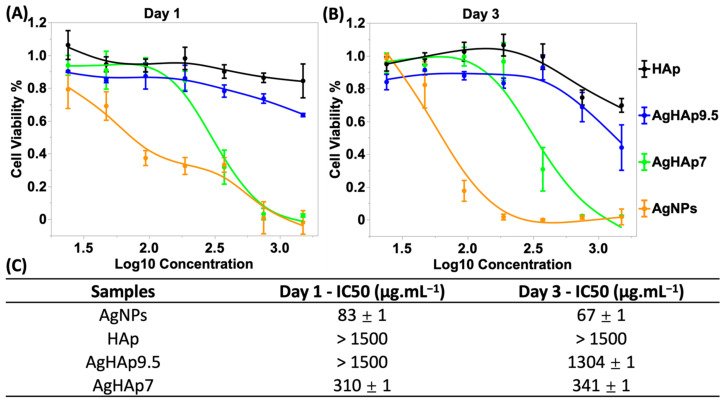
Fibroblast L-929 cell viability by MTT assay on Day 1 (**A**) and Day 3 (**B**) of exposure to various NPs at different concentrations. (**C**) IC50 values of AgNPs, HAp, AgHAp9.5, and AgHAp7. Curves indicate fits (see Section 2) to the experimental data (solid circles): AgNPs (orange), HAp (Black), AgHAp9.5 (Blue), and AgHAp7 (Green). IC50 values were obtained from curve fits.

Cellular metabolic activity was determined by the MTT assay as an estimate of cell viability (Figure 6A,B). Fibroblast viability was significantly lower on Day 1 (Figure 6A) and Day 3 (Figure 6B) for cells treated with Ag and AgHAp7 NPs compared to AgHAp9.5 and pure HAp NPs. Quantitation of the cytotoxicity was conducted by determining the IC50 value, at which 50% of the cell population is metabolically viable (Figure 6C). In detail, curves fit to the IC50 data showed that AgNPs exhibited greater cytotoxicity than AgHAp7 NPs, consistent with the LIVE/DEAD staining results (Figure 5). By Day 3, however, the cell viability in the presence of AgNPs plunged significantly at a concentration of 94 μg.mL^−1^, and nearly all the cells were unviable at 188 and 375 μg.mL^−1^ (Figure 6C). Viability also decreased by Day 3 with AgHAp7 treatment, but the drop was more gradual, and major cytotoxicity was observed at concentrations ≥ 375 μg.mL^−1^. The IC50 for AgNPs was much higher than IC50 for AgHAp7 at Day 3 (Figure 6C). AgHAp9.5 and pure HAp NPs had similar cytotoxicity trends with concentration and time, but AgHAp9.5 was slightly more toxic than HAp.

In summary, the viability assays and antimicrobial tests confirmed that AgHAp7 is the least cytotoxic and most efficacious antimicrobial material studied here against *F. nucleatum* and *S. mutans* compared to AgNPs.

## 4. Discussion

### 4.1. AgHAp NPs Compared to AgNPs

We demonstrated here that silver is present as Ag^+^ ion in the bulk HAp crystal structure. This is significant, because Ag^+^ is the direct antimicrobial agent [15,16,22,55]. In contrast, the AgNP surface requires oxidative dissolution, a process in which surface Ag^0^ atoms have to be oxidized to Ag^+^ and only then can they be released to a solution [15,16,17,18,19]. This process is thermodynamically unfavorable under anaerobic conditions [20,21]. Thus, AgHAp NPs were shown to be more efficacious than standard AgNPs as a broad-spectrum antimicrobial material against anaerobic and aerobic oral bacteria and more biocompatible to fibroblasts cells. The poor antimicrobial efficiency of AgNPs against *S. mutans* may be related to the kinetically slow rate of Ag^+^ release, possibly due to the presence of a thin surface layer of Ag_2_O.

In addition to the silver oxidation state, the particle size and surface area of NPs may also control the antimicrobial efficacy. For example, reducing the size of silver nanoparticles to ~5–10 nm can enhance their antimicrobial efficiency against both aerobic and anaerobic bacteria [21,77,78,79], but it can also lead to significantly higher cytotoxicity [80]. In another study, the synthesis of high surface area AgHAp NPs (~200 m^2^/g) with low Ag content (<0.5 wt %) stabilized the formation of Ag_3_PO_4_ NPs (<3 nm) [81]. Those authors suggested that Ag_3_PO_4_ NPs can release Ag^+^ rapidly to inhibit aerobic Gram-negative and Gram-positive bacteria, *P. aeruginosa* and *S. Aureus*, respectively.

### 4.2. Synthesis pH-Dependent Tunability and Tissue-Specific Applications

We showed that the Ag content of HAp NPs is tunable by an inverse relationship to synthesis pH [47]. This simple yet powerful control of the Ag content suggests that HAp NPs can be used to inhibit bacteria of different susceptibilities, depending on the specific tissue application. In the present study, AgHAp7 NPs emerged as the material with the optimum balance between antimicrobial efficacy against oral bacteria and murine fibroblast viability compared to AgHAp NPs synthesized at higher pHs. We have previously demonstrated that AgHAp9.5 NPs were optimally antimicrobial against the most common orthopedic bacteria, *E. coli* and *S. epidermidis*, and biocompatible with murine pre-osteoblasts [47] compared to AgHAp11 NPs.

### 4.3. Antimicrobial Resistance

A potentially significant advantage of our AgHAp NPs over antibiotics and AgNPs is that of a lower probability for the development of antimicrobial resistance. Antimicrobial resistance may arise due to at least two reasons, namely, the presence of foreign solid NPs and the release of soluble Ag^+^ ions. Each of these potential causes of antimicrobial resistance, as well as their applicability to *F. nucleatum* and *S. mutans*, are discussed below.

Recent research reveals that some types of pathogens can develop resistance to nanoparticles, including AgNPs. For example, aerobic Gram-negative *E. coli* and Gram-positive *P. aeruginosa* produce flagellin, an adhesive protein in the bacterial flagellum, which causes the aggregation of AgNPs, thus resisting the effects of AgNPs [17,24,82]. However, both *S. mutans* and *F. nucleatum* lack flagella, so this mechanism of antimicrobial resistance to AgNPs or AgHAp NPs is not likely [83,84]. To our knowledge, there are no published reports that have examined whether *F. nucleatum* develop antimicrobial resistance to AgNPs.

We next consider the potential development of antimicrobial resistance against dissolved silver ions. While there is no known bacterial silver ion efflux pump, there does exist a “*sil* operon” in the Gram-negative facultative anaerobes *Salmonella typhimurium*, *Klebsiella pneumoniae*, and *Enterobacter hormaechei*. The *sil* operon codes for silver-binding proteins. It has been conjectured that this operon may also code for silver efflux pumps [85], but this is pure conjecture, and it has never been shown to occur [82], so we deem this unlikely. To our knowledge, there are no reports that *F. nucleatum* and *S. mutans* possess *sil* operon-related genes. Another Gram-negative facultative anaerobe, *E. coli*, which lacks the *sil* operon, does not develop resistance to Ag^+^ ions, while it does develop resistance to AgNPs [17]. Based on the current state of knowledge, therefore, we cautiously suggest that the bacteria studies in the present study will not develop antimicrobial resistance to soluble Ag^+^ ions.

### 4.4. Cost-Effectiveness, Scalability, and Medical Use

The AgHAp NP synthesis method is easy and cheap, and therefore, it is imminently scalable. The AgHAp NPs may be used, for example, as coatings on wire brackets of braces or in pouches for periodontal applications or as surface coatings on orthopedic implants. These NPs also have a pleasing white color compared to the black color of AgNPs, which would increase usage compliance of the treatments by patients.

### 4.5. MIC Tests for Strictly Anaerobic Fastidious Bacteria

A significant finding of our study is that the MIC is strongly affected by the inclusion of reducing agents like L-Cys, which is critical for fastidious anaerobic bacteria, especially those associated with periodontitis, such as *F. nucleatum* and *Campylobacter jejuni* [86,87,88]. If not included, these bacteria will be highly susceptible to any new antimicrobial being tested, thus yielding a spuriously low MIC. On the other hand, if L-Cysteine is included, it can complex Ag^+^ or other antimicrobial metals such as Zn^2+^, Cu^2+^, etc., which reduces the ion concentration, yielding a spuriously high MIC value. Unfortunately, many studies do not specify whether they employed L-Cys or other reducing agents. The inhibition zone test does not suffer from the potential limitations of the MIC test, so we recommend the former as a more reliable antibacterial test against fastidious anaerobic bacteria. Further, the comparison of MIC values across the literature is also hindered by a lack of methodological details in some studies, such as the initial bacterial concentration employed.

## 5. Conclusions

We synthesized Ag-hydroxyapatite nanoparticles (AgHAp NPs) by the wet precipitation method at different pHs. The Ag content varied inversely over an order of magnitude (4.5–0.45 wt %), with the synthesis pH (6–11). We showed that AgHAp NPs synthesized at pH 7 (AgHAp7) have the optimum balance of low cytotoxicity against murine fibroblast cell line L929 on the one hand and broad spectrum, tunable, antimicrobial ability on the other hand against *F. nucleatum*, a fastidious, anaerobic Gram-negative bacterium in strictly anaerobic medium and, also, against *S. mutans*, an aerobic (facultative anaerobe) Gram-positive bacterium. The antimicrobial ability and cytotoxicity of AgHAp7 NPs is better than that of standard AgNPs. This greater effectiveness of AgNPs is because, as proven here by XPS, these NPs contain Ag^+^ ions, which are the antimicrobial agent in HAp and can directly release Ag^+^ ions even under anaerobic conditions compared to Ag^0^ in AgNPs. Importantly, *F. nucleatum* and *S. mutans* may be less likely to develop antimicrobial resistance to Ag^+^ ions than to AgNPs. Moreover, the pH-dependent Ag^+^ content of AgHAp NPs provides an easily tunable parameter for antimicrobial applications in different tissues, including teeth, gums, and bone. For antimicrobial testing of fastidious anaerobic bacteria, the inhibition zone test is a preferable choice over MIC tests. The ease of synthesis and scalability offer an inexpensive pathway to relieve the suffering of a large fraction of the global population worldwide afflicted by various oral infections, which can develop into more serious chronic and even potentially fatal conditions. 

## Figures and Tables

**Table 1:** Chemical characterization of AgNPs and AgHAp NPs by XPS. Atomic composition (wt %) obtained from the XPS survey scan (Figure 2A). Ag wt % obtained by converting atom % from XPS data.

Nanoparticle	Stoichiometric Formula	XPS Survey Scan					
		Ca	P	O	C	Ag	Ag
	Ca_10−x_Ag_x_(PO_4_)_6_(OH)_2−x_	(atom %)					(wt %)
AgNPs	-	-	-	16.6	60.7	22.7	-
AgHAp9.5	Ca_9.97_Ag_0.03_(PO_4_)_6_(OH)_1.77_	16.1	11.5	56	16	0.5	2.52
AgHAp7	Ca_9.95_Ag_0.05_(PO_4_)_6_(OH)_0.71_	15.7	12.4	56.8	14.2	0.9	4.43

**Table 2:** Oxidation state characterization of AgNPs and AgHAp obtained by a XPS high-resolution (HR) scan of the Ag3d_5/2_ peak (Figure 2B). The % of ionic silver was calculated from fitting the Ag3d_5/2_ peak.

Fitted Area under Ag3d5 Peak	Ag^+^ (367.7 eV)	Ag^0^ (368.8 eV)	Ag^0^ + Ag^+^	Ag^+^/(Ag^0^ + Ag^+^) %
AgNPs	779	11,931	12,710	6%
AgHAp9.5	992	203	1195	83%
AgHAp7	565	88	653	87%

**Table 3:** MIC values (μg.mL^−1^) of AgNPs, AgHAp9.5, and AgHAp7 against *F. nucleatum* and *S. mutans* in various media. MIC represents the whole NP concentration, while Ag MIC is the whole NP concentration normalized by the silver content obtained from ICP-OES analysis.

Nanoparticle	*F. nucleatum*; Medium 1 ^a^		*F. nucleatum*; Medium 2 ^b^		*S. mutans*; Medium 3 ^c^	
	MIC	Ag MIC	MIC	Ag MIC	MIC	Ag MIC
AgNPs	>2000	>2000	1500	1500	>1000	>1000
AgHAp9.5	>2000	>35	1000	18	1500	26
AgHAp7	1500	63	375	16	375	16

^a^ Medium 1: Brain–heart infusion broth with yeast extract, vitamin K1, hemin, and L-Cys. ^b^ Medium 2: identical to Medium 1 but without L-Cys. ^c^ Medium 3: only brain–heart infusion broth (see Section 2 for details).

## Data Availability

The original contributions presented in the study are included in the article/Supplementary Material, further inquiries can be directed to the corresponding authors.

## Supplementary Materials

The following supporting information can be downloaded at: https://www.mdpi.com/article/10.3390/ma17194688/s1. Figure S1: The color and appearance for the NPs; Figure S2: Particle Size Distribution Obtained by TEM Analysis; Figure S3: Growth Media Effects on MIC of AgNPs and AgHAp NPs against F. Nucleatum and S. Mutans; Table S1: Total Silver content in each silver-doped hydroxyapatite nanoparticle (AgHAp NP) type; Table S2: Diameters of Inhibition Zones.
