# Supplementary material for: Direct, Broad-Spectrum Antimicrobial Activity of Ag+-Doped Hydroxyapatite against Fastidious Anaerobic Periodontal and Aerobic Dental Bacteria
Source: Materials (Basel). 2024 Sep 24;17(19):4688. doi: 10.3390/ma17194688 (PMC11478222; doi:10.3390/ma17194688)
Supplement: Supplementary file 1 [file materials-17-04688-s001.zip › materials-3127294-supplementary.pdf]

Supporting Information for Publication

Date: 09/24/2024

For Submission to: Materials

Direct, Broad-Spectrum Antimicrobial Activity of Ag^+-Doped Hydroxyapatite against Fastidious Anaerobic Periodontal and Aerobic Dental Bacteria

Ruibo Hu [1], Leyi Deng [1], Xiaoying Hao [2], Jiadong Chen [1], Xianfeng Zhou [2,] and Nita Sahai [1,3,4,5,*]*

[1] School of Polymer Science and Polymer Engineering, The University of Akron,
Akron, OH 44325-3909, USA

[2] College of Material Science and Engineering, Qingdao University of Science and Technology,
Qingdao 266042, China

[3] Integrated Bioscience Program, The University of Akron, Akron, OH 44325-3909, USA

[4] Department of Geosciences, The University of Akron, Akron, OH 44325-3909, USA

[5] Department of Biology, The University of Akron, Akron, OH 44325-3909, USA

* Correspondence: xianfeng@qust.edu.cn (X.Z.); sahai@uakron.edu (N.S.)

Number of pages: 5

Number of figures: 3

Number of tables: 2

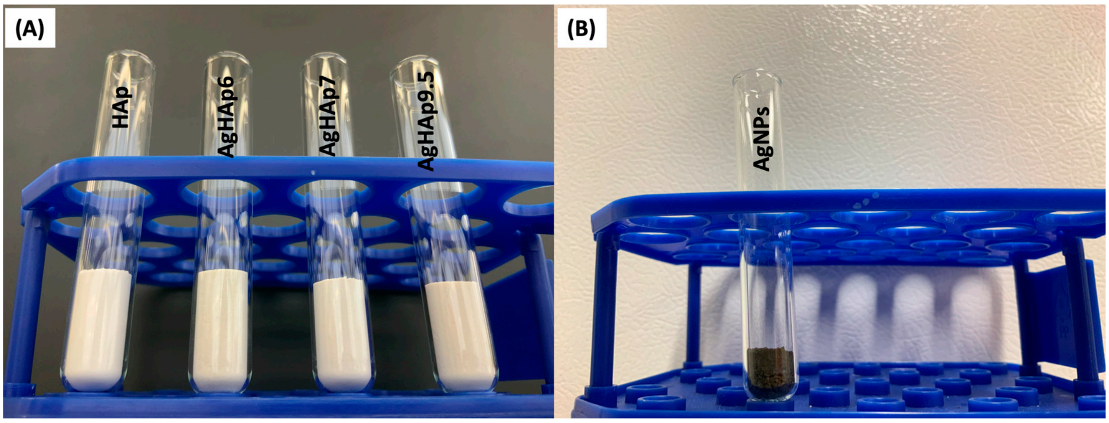

Figure S1. The color and appearance for the NPs. (A) HAp, AgHAp6, AgHAp7, andAgHAp9.5 from left to right and (B) AgNPs.

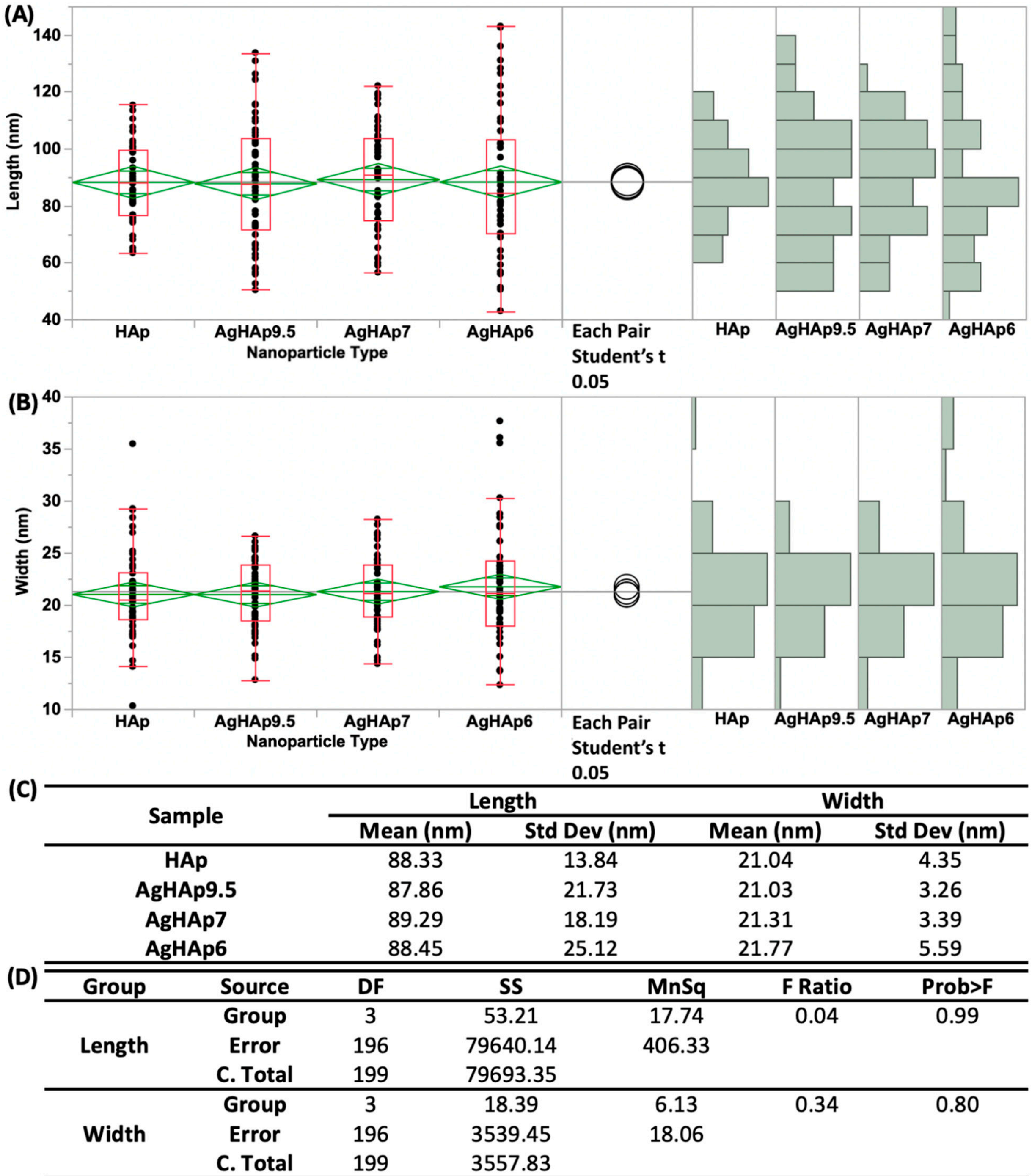

(C)

Sample	Length		Width	
	Mean (nm)	Std Dev (nm)	Mean (nm)	Std Dev (nm)
**HAp**	88.33	13.84	21.04	4.35
**AgHAp9.5**	87.86	21.73	21.03	3.26
**AgHAp7**	89.29	18.19	21.31	3.39
**AgHAp6**	88.45	25.12	21.77	5.59

(D)

Group	Source	DF	SS	MnSq	F Ratio	Prob>F
**Length**	**Group**	3	53.21	17.74	0.04	0.99
	**Error**	196	79640.14	406.33		
	**C. Total**	199	79693.35			
**Width**	**Group**	3	18.39	6.13	0.34	0.80
	**Error**	196	3539.45	18.06		
	**C. Total**	199	3557.83			

Figure S2. Particle Size Distribution Obtained by TEM Analysis: (A) Length and (B) Width. The size of each sample with corresponding ANOVA statistical analysis in (C) and (D), respectively. Abbreviations: Std Dev = Standard deviation; DF = Degree of freedom; SS = Sum of squares; Mn Sq = Mean Square; C. Total = Counting total.

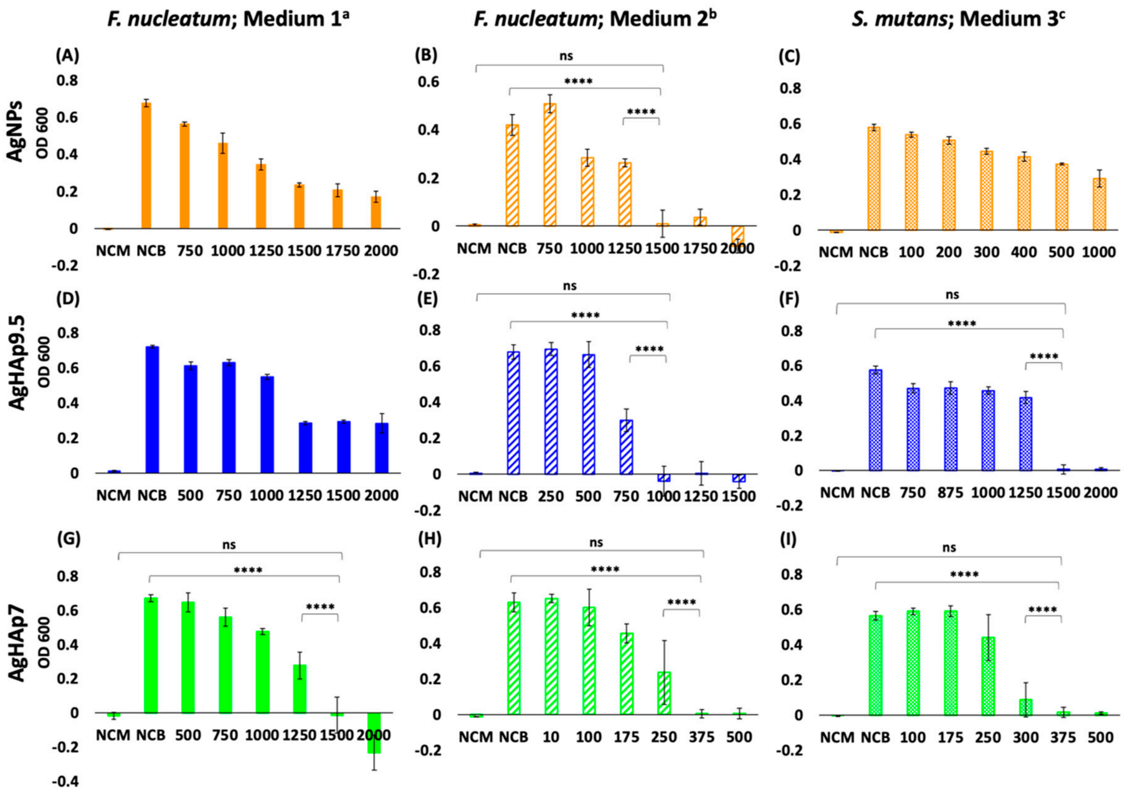

Figure S3. Growth Media Effects on MIC of AgNPs and AgHAp NPs against *F. Nucleatum* and *S. Mutans*. Orange bars: AgNPs; blue bars: AgHAp9.5 NPs; and green bars AgHAp7 NPs. (A), (D), (G): Solid bars refer to *F. nucleatum* in Medium 1 consisting of Brain Heart Infusion Broth with Yeast Extract, Vitamin K1, hemin, and L-Cys.; (B),) (E), (H): hatched bars refer to *F. nucleatum* in Medium 2[b], which is the same as Medium 1 but without L-Cys; and (C), (F), (I): checkered bars refer to *S. mutans* in Medium 3 consisting of Brain Heart Infusion Broth (see Methods section for details). NCM represents Negative Control Medium (Pure Medium) and NCB meaning that Negative Control Bacteria (Medium + Bacterial Stock). Student's t-test was utilized for determining MIC (∗∗∗∗ < 0.0001, ns = no significant difference compared to NCM).

Table S1. Total Silver content in each silver-doped hydroxyapatite nanoparticle (AgHAp NP) type.

AgHAp NP	Synthesis pH						
	**6.00**	**7.00**	**7.50**	**8.00**	**8.50**	**9.50**	**11.00**
Silver Content (wt%)	4.17	4.17	4.35	4.12	3.74	1.76	0.45
Std Dev	0.07	0.21	0.09	0.06	0.10	0.03	0.02

Table S2. Diameters of Inhibition Zones: Inhibition Zone diameters obtained using AgNPs, AgHAp9.5, AgHAp7 and AgHAp6 NPs against *F. nucleatum* and *S. mutans*.

Types of AgHAp	Bacterium	Growth Condition	Diameter (mm)
AgNPs	*F. nucleatum*	Anaerobic	7.02 ± 0.29
AgHAp9.5	*F. nucleatum*	Anaerobic	7.56 ± 0.40
AgHAp7	*F. nucleatum*	Anaerobic	10.48 ± 0.90
AgHAp6	*F. nucleatum*	Anaerobic	10.63 ± 0.73
AgNPs	*S. mutans*	Aerobic	7.01 ± 0.27
AgHAp9.5	*S. mutans*	Aerobic	8.39 ± 0.42
AgHAp7	*S. mutans*	Aerobic	10.40 ± 0.29
AgHAp6	*S. mutans*	Aerobic	10.36 ± 0.23
